# Cooling Island Effect of Blue-Green Corridors: Quantitative Comparison of Morphological Impacts

**DOI:** 10.3390/ijerph182211917

**Published:** 2021-11-13

**Authors:** Yunfang Jiang, Jing Huang, Tiemao Shi, Xiaolin Li

**Affiliations:** 1 School of Urban and Regional Science, East China Normal University, Shanghai 200241, China; 51193902030@stu.ecnu.edu.cn (J.H.); 51203902026@stu.ecnu.edu.cn (X.L.); 2 The Center for Modern Chinese City Studies, East China Normal University, Shanghai 200241, China; 3 Research Center for China Administrative Division, East China Normal University, Shanghai 200241, China; 4 Institute of Spatial Planning and Design, Shenyang Jianzhu University, Shenyang 110168, China

**Keywords:** urban heat island (UHI), blue-green space, spatial morphology, urban cooling island (UCI), boosted regression trees (BRT), China

## Abstract

The patterns of green corridors in urban riverfront districts provide different synergistic cooling effects of blue-green space in urban areas. The purpose of this study is to quantify the spatial morphological impact of green corridors in riverfront block-scale area on the cooling effect. Three representative patterns (radiate, grid and dendritic) were selected in the study. The comprehensive influences analysis between multi-dimensional factors of spatial structure and morphology of green corridors and Ta (air temperature) distribution are processed by Envi-met4.4.5 simulation data and statistical analysis methods, such as regression tree model (BRT), were combined. The results showed that the D (distance from riverbank) has the greatest impact on the cooling effect of each belt green space. The D in the range of 600–750 m was affected by the cooling effect of blue-green space; The orientation with parallel to (southeast–northwest) or roughly the same as the prevailing wind direction (north–south) green corridors had relatively better cooling effect. When the width of green corridor was 20–25 m, the ME (marginal effect) of cooling was the largest; at 30–35 m (corridor width), the overall ME of cooling was the best; When the dPC (decreased probability connectivity, here the index was adapted to describe the connectivity degree) of green corridors was in the range of 0.5–1.5, the cooling effect of green corridor could be significantly improved. When dPC is 1.5, its marginal effect on temperature reached the maximum. The study provided a quantitative correlation technology for the morphological influence of blue-green space on the distribution of UCI (urban cooling island), which can guide the spatial layout control of green corridors in the planning and design of urban riverfront district.

## 1. Introduction

Urban blue-green spaces composed of water bodies and green spaces in the city can cool the air temperature in the surroundings of built-up areas and produce urban cooling islands (UCIs). Urban green spaces (UGSs) can regulate the surface energy exchange processes through evapotranspiration, shadowing, emissivity adjustment and air movement and heat exchange effects [1,2,3] and often form UCIs during hot weather periods [4,5]. Urban blue infrastructure can most effectively emit longwave radiation to cool the surface because it has high emissivity and efficiently consumes shortwave radiation through evaporation. At the same time, water surface energy can be transferred through conduction, convection and advection with water bodies [1,6,7]. The combination of waterbodies and green spaces produces a good synergistic cooling effect [8].

The synergistic cooling effect of the blue-green space is considerably higher than that of any single ecological element [9,10,11,12]. It is found that vegetation can affect the radiation balance of water body and adjust the water temperature through shading, so as to promote air convection. It is pointed out that high-density vegetation can reduce the riparian temperature, which is verified by a model for vertical turbulent diffusion and stratification in a shallow lake study [13]. The cooling effect of waterfront green corridor widths above 60 m is much greater than that of 30–60 m [14]. The cooling effect of vegetation-covered riverbanks is higher than that of hard-ground riverbanks [15]. The spatial structure of waterfront vegetation also produces different blue-green synergistic cooling effects. The higher the canopy density of the vegetation cover, the less the solar radiation and the lower the temperature [11]. The blue space with at least width 20 m could further enhance the cooling island and the cold air diffusion channel and ventilation corridor by green space network can strengthen the cooling effect to a certain extent [16].

From the perspective of the cooling impact of rivers’ spatial pattern, it is mainly affected by the distance between the waterbody and the green space as well as the orientation and width of the river [17,18]. The case study of an urban region in Fujian indicated that green spaces adjacent to rivers can exert obvious synergistic cooling effect, which can intensify the cooling effect approximately 2.7 °C [19]. The results of field observations on the micro-climate in and around the Ota River flowing through Hiroshima City in Japan Showed that the thermal effects were discernible at least a few hundred meters horizontally and more than 80 m vertically [18]. The distance to the river is more important that the urban score at a station for predicting the UHI. For every 1000 m increase in distance from the river, the UHI decreased by 0.6 °C in the summer [20]. The downwind area relative to the river can obtain a cooling intensity higher than 1.5 °C compared to the upwind area [21]. A river with a width of 35 m can lead to a decrease of approximately 1–1.5 °C in the ambient temperature and the decrease can be increased if green space if present on both sides of the river [22]. The larger the river width, the stronger the ability to alleviate the thermal environment [23].

The specific spatial factors of green space influencing the cooling effect usually include the area, green space coverage and spatial configuration [24,25,26]. Green patches with large areas can provide significant and stable cooling [27,28,29]. Within a certain range, increasing the green patch area leads to a significant improvement in its cooling effect. However, there is the threshold value of efficient (TVoE) according to the “law of diminishing marginal utility” in economics [28]. Once its area exceeds the threshold value, the increase in the cooling effect is very small. The area of tree-based green space within approximately 0.32 ha can effectively enhance their cooling intensity [30]. A high coverage ratio of green space corresponds to a lower surface temperature [31,32]. Belt-shaped green areas parallel to the wind direction appear to have a higher cooling effect than those vertical to the wind direction and the difference of the cooling effect reached 6.3 K between the two orientation scenarios with big canopy trees [33]. Although the cooling island intensity of a belt park is relatively small, it has a large diffusion distance from the cooling island [19]. In addition, green spaces with higher connectivity degrees improve the cooling effect over adjacent areas [16,34,35]. A strong positive relationship exists between the cooling capability of urban river wetlands and the hydrologic connectivity of river wetlands based on a case study in cities of northeast China [17]. The sky view factor (SVF) is also important in morphology-related urban microclimate studies. SVF values ranging from 0.2 to 0.4 reduce the air temperature by approximately 0.3 °C for urban greenery, improving the outdoor thermal environment [30].

Different physical surface material of built environment produce radiant heat transfer and convective heat transfer, which are important factors causing urban heat island. From the perspective of radiation heat transfer, albedo changes the reflected heat and heat absorption; at the same time, from the perspective of convective heat transfer, the thermophysical properties of different materials in the built environment have changed, resulting in a large air temperature difference between the surface temperature of artificial materials and water containing materials such as vegetation and water, which further increases the output of convective heat transfer between them [36]. The different spatial element components in a green space affect the albedo. An increase in the surface albedo (S_albedo_) reduces the surface temperature [37] and affects the temperature distribution of the green space and its surrounding areas. More widely, the literature state that the S_albedo_ generally has a positive proportional relationship with the Ta [38,39]. An increase in S_albedo_ from 0.5 to 0.8 led to variation in the ground surface temperature (T_surface_) by some 5 °C [40]. The difference in surface environment caused by differences in the green space enclosure edges is also an important factor influencing the microclimatic effect [28,41]. The cooling island intensity of a park may also be influenced by the specific characteristics of the surrounding environment. In addition, the variation in the thermal properties of different types of built surfaces, such as asphalt roads and building roofs, can contribute to surface temperature variations in surrounding non-park environments [42]. There is a negative relationship between the cooling capability of urban river wetlands and height and density of surrounding buildings [17]. The average T_surface_ of natural green spaces around urban built-up areas is generally 1–2 °C lower than that of various artificial green spaces distributed in such areas [43]. The more concentrated the green space distribution, the higher the intensity of the cooling island effect [44].

Based on the above-mentioned studies, there has been extensive and quantitative research on the impact of green space structural and morphological factors on the microclimate. However, the synergistic cooling effect of blue-green space complex systems only focuses on two impact factors of spatial morphology, namely, the belt green space width in the riverfront area and vegetation coverage [11,12,13,14,15]. In addition, the spatial structure and morphology influences of green corridors connected with the riverside green corridor are not considered in the synergistic cooling effect as a holistic system [16,45]. The riverside green corridor should not only be perceived as an isolated public landscape space. Based on this holistic system perspective, in a particular range of urban riverfront districts, scientific and suitable organisation of blue-green corridor systems is necessary to enhance the overall microclimatic effect. Our study aims to determine what specific type of spatial pattern of blue-green corridors maximises the cooling effect in hot diurnal periods. Furthermore, we intend to investigate how to combine quantitative morphological and structural index controls to provide the maximum cooling effect of belt green spaces, so that we can offer green pattern guidance for climate adaptability planning in the waterfront region.

To achieve the aforementioned aims, three spatial structural patterns of green corridors in urban riverfront districts, which have clear differences in terms of corridor orientation, width and connection characteristics, were selected. Based on the basic data obtained from the dynamic microclimate simulation software ENVI-met 4.4.5, the common impact effect of the spatial structure and morphology index of green corridors on the air temperature distribution was quantitatively analysed. The correlation between the spatial index and cooling effect of the blue-green space is not necessarily linear. Machine learning algorithms have the advantage that they do not require presetting of the relationship model in correlation research; Thus, new insights on the correlation between factors can be obtained. The boosted regression tree (BRT) model, a type of machine learning algorithm model, was used to analyse the contribution ratio and importance of various spatial factors in the cooling effect aspect under multiple interactions between the structural and morphological factors of the riverside green corridor. Furthermore, the BRT model was used to analyse the threshold values of the marginal effect (ME) and influence characteristics of each morphological factor on the temperature distribution of the riverside green corridor. This research can provide basic control methods and layout suggestions for optimising the climate adaptability of green space patterns in metropolitan waterfront areas.

## 2. Study Area and Methodology

### 2.1. Study Area

Shanghai is located at 31°12′ N latitude and 121°30′ E longitude with a subtropical monsoon climate. It is hot and humid in summer and the average temperature has continued to rise in the past 10 years with the prevailing southeast wind. It has sufficient precipitation, rich water resources and a dense river network. Numerous river systems provide a large number of leisure activities shared by all citizens. In recent years, Shanghai has made full use of the current urban river resources and restored the green space along the riverside to form an interconnected blue-green network and public space with amenities. The ecological cooling effect of the blue-green landscape space makes it an important feature directly related to urban living comfort, energy conservation and low-carbon emissions.

**Figure 1:**
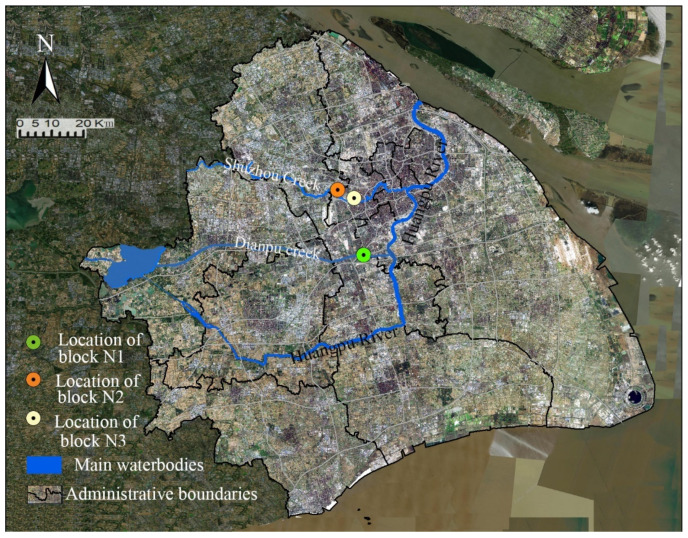
Location of the three study areas in Shanghai, China.

**Figure 2:**
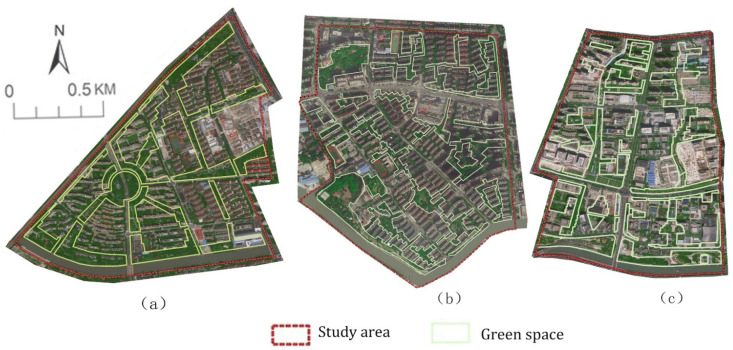
Satellite images and green space patterns of the three study areas in 2019: (**a**) block 1 (N1), (**b**) block 2 (N2) and (**c**) block 3 (N3).

Three study areas (blocks) with almost the same district coverage were selected. The rivers pass by southwards and they are all located in the upwind direction of the selected district blocks to make the main green corridor connected to the river achieve a greater cooling effect. The river across the south of block N1 is Dianpu creek and block N2 and block N3 were selected on the North riverbank of Suzhou creek. Both Dianpu River and Suzhou River are important rivers in Shanghai and the river segment width is set as approximately 56 m (Figure 1). Moreover, there are complete green corridors connected to the river section. The research scheme was designed such that the coverage ratio of the green space and plant configuration is the same in the three areas. The vegetation configuration was combined with a unified arbour forest and grassland and the ratio of arbour to grass was 1:30 plants/m^2^. The buildings were arranged in a determinant style with approximately the same development intensity. To reduce serious interferences from other open spaces, e.g., the road network, on microclimate, a loamy soil blankets was used in the modelling design and low-rise buildings within the controlled boundary of the green corridors were also configured as green vegetation coverage areas. Eventually, three case study blocks with typical green corridor systems were identified (Figure 2).

For the first block, the radiating pattern of green corridors with a typical regular layout was selected. In contrast, for the second block, the latticed grid pattern of green corridors was chosen. Finally, the third block used the dendritic pattern with south–north extension green corridors. The detailed conditions of the three study blocks are as follows.

Block N1 covers an area of 1.43 km^2^ with a green space ratio of 27.69%. The building density is about 36.85%,which was composed of multistorey (with the building height from 18 m high to 21 m) and small high-rise residential buildings (with the building height of 33 m and 45 m), surrounded by public multifunctional buildings. The area has a radiating layout of green spaces and the central area is a circular green space, which is a radially interspersed corridor green space with different orientations in each building area. The width of the green corridors varies from 12 m to 50 m and a few connected green corridors are distributed between them, forming an interconnected green space network layout as a whole. The width of the riverside green corridor in the south varies from 60 m to 100 m (Figure 2a).

Block N2 covers an area of 1.44 km^2^ with a green space ratio of 28.48%. The building density is about 34.90%, which is planned as the same styles as the block N1 to reduce the thermal interference of built environment. The dominant orientation of green corridors in this area is mainly from southeast to northwest, extending to both sides to form the southwest–northeast branch perpendicular to the main green corridors, which finally presents a latticed grid green space layout in the south and middle of the block. The green space network in this area is evenly distributed and interspersed in each building group. The width of most riverside green corridors is narrow, with its width about 35 m. A larger riverside green space with a width of approximately 95 m is distributed in the southwest of the river (Figure 2b).

Block N3 covers an area of 1.34 km^2^ with a green space ratio of 27.86%. The building density is about 33.15%, which is also arranged the same group of building height as the block N1 and block N2 to reduce the thermal interference of built environment. The area is mainly a north–south green corridor layout with a small number of narrow green branches and different sizes of green patches, with a dendritic pattern. There is also a wide riverside green corridor in the south of the block, most of which are 90–135 m in width. A small section is occupied by the internal buildings of the green space and the narrowest width is approximately 55 m (Figure 2c).

### 2.2. Variables of the Study

#### 2.2.1. Multidimensional Spatial Variables

The blue-green spatial systems with different characteristics in the case area could result in various air temperature distributions. The distribution of distance from the riverbank (D), orientation (O), connectivity degree (here adapted as decreased probability connectivity, abbreviated as dPC), green corridor area (GA) and width (GWd) and SVF of each green patch, the surface albedo (S_albedo_) and the ground surface temperature (T_surface_) were concerned. These are the main controlling factors on the cooling effect. Combined with these spatial configurations of the system properties, the quantitative indices of their spatial structure and morphology were partitioned into three aspects, namely the structural factors of blue-green space, spatial morphology and environmental factors of green corridors (Table 1).

The structural factors of blue-green space present the holistic structural relationship of the waterfront green corridor system. In this study, three indices were selected, i.e., distance from the riverbank, orientation and dPC. The spatial morphology factor of green corridors was used to describe the three-dimensional (3D) morphological characteristics of each specific green space, including the area (GA), width (GWd) and SVF of each green corridor segment. The environmental factors of green corridors were selected to reflect the influence of the built environment around the green corridors and the spatial layout difference inside the green corridor on the air temperature (Ta) of each green corridor segment, e.g., surface albedo (S_albedo_) and surface temperature (T_surface_). The two environmental indices of the green corridor can comprehensively reflect the differential cooling effect of the green corridor caused by the surrounding conditions arising from the diversity of green space agglomeration, surrounding building enclosures and internal space elements (Table 1).

Regarding the selection of the spatial morphology index system, it should be noted that there are many indices influencing the Ta of green corridors in previous research, such as LSI, coverage ratio of green space and spatial 3D green quantity. In the studies of [1,16,19,24,33], the coverage ratio of the green space and 3D green quantity was controlled so that it is almost the same as the unified layout criterion in the study design process. Meanwhile, the three blocks were all located in urban built-up areas and the LSI values of green patches were partitioned regularly and changed slightly. It is not used as a meaningful spatial index of green corridors to describe the differences in morphological factors.

Another aspect that needs to be explained is that although the same configuration criteria of green space layout are adopted in the simulation scheme to uniformly plan and arrange the vegetation composition, spatial layout in the green space and construction intensity of surrounding buildings in three blocks, the surrounding and internal spatial environmental differences still need to be considered. The T_surface_ index of each green space reflects the differences in the space elements, such as soft and hard ground, as well as the vegetation coverage inside the green space. The differences in solar radiation changes of various artificial green spaces present the T_surface_ value differences. S_albedo_ is also related to the reflectivity and enclosure of building surface materials around the green corridors, as well as the sun reflection differences of vegetation shading, leaf surface and transpiration within the green space, resulting in differences in the S_albedo_ in each green space.

The values of the SVF, S_albedo_ and T_surface_ of green corridors were obtained from the surface data through the ENVI-met 4.4.5 (ENVI-met GmbH, Essen, Germany) simulation results. The width and area values of green corridors, both of which were used to describe the scale of each green corridor, were obtained by direct measurement and calculation based on the simulation model transferred to ArcGIS (Esri, Redlands, CA, USA). The quantitative calculation of the other three indices is described as follows:

(1) Distance from the riverbank (D)

The data management tools in ArcGIS 10.4 were used to extract the geometric centre coordinate points of each green space via the tools for feature to point and then the near distance analysis in the analysis tools was used to calculate the shortest distance from a point to a line. Eventually, the shortest distance from the geometric centre of each green space to the north bank of the river was obtained.

(2) Orientation value assignment

The green corridor was divided into four orientations: east–west (E–W), south–north (S–N), southwest–northeast (SW–NE) and southeast–northwest (SE–NW). The influence of the different orientation types of green corridors on the Ta distribution was analysed. Since this index is a categorical variable, in the correlation analysis between this index and Ta, the orientation values were assigned as follows: 1 = E–W, 2 = S–N, 3 = SW–NE and 4 = SE–NW.

**Figure 3:**
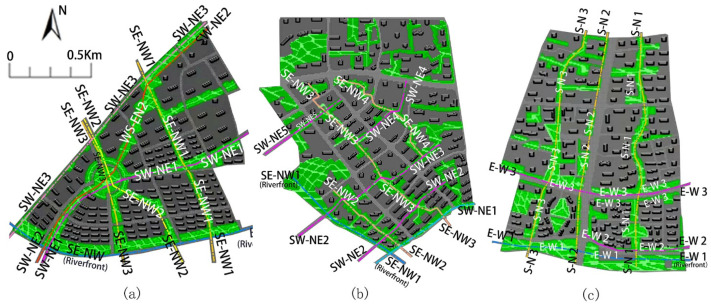
Simulation modelling and schematic of the main green corridors with different orientations in the three study areas: (**a**) block N1, (**b**) block N2 and (**c**) block N3. Note: The simulation model extracts the main spatial structure of the selected riverfront block and combined the urban design method to control the specific factors influence for feasible comparative study.

The green corridors with different orientations were identified and determined according to their integrity and importance in the study areas (Figure 3). Block N1 has multi-axial green corridors with a radiating pattern. There are riverside green corridors containing two orientations (E–W and SE–NW), three green corridors with an SW–NE orientation and three green corridors with an SE–NW orientation (Figure 3a). Block N2 has two vertically oriented green corridors, i.e., SE–NW and SW–NE orientations, interweaving to a typical green corridor pattern. It also has riverside green corridors with two orientations (SE–NW and SW–NE (Figure 3b)). In contrast, block N3 has three main green corridors with an S–N orientation, combined with an E–W riverside green corridor with a partial E–W orientation to form a dendritic green corridor pattern (Figure 3c). Among these types of corridor orientations, the SE–NW and S–N orientations have an angle of zero (or acute) during the summer monsoon, which is parallel to the prevailing summer wind (southeast wind).

(3) Decreased probability connectivity

The degree of connectivity is an effective index for evaluating the continuity of the landscape spatial structure. The dPC (decreased probability connectivity) index was selected to evaluate the degree of connectivity of the green space in the entire blue-green ecological network and measure the influence of the connectivity of the green space. The threshold distance of the connectivity was set as 50 and the probability of connectivity of patches was set as 0.5 (Saura and Pascual-Hortal, 2007). The Conefor Sensinode 2.6 software was used to calculate the dPC value of each green space as follows: First, the probability of connectivity (*PC*) was calculated and the dPC values were calculated based on the *PC*. The calculation formulas are given by Equations (1) and (2):

(1) $$PC=\frac{\sum_{i=1}^{n}\sum_{j=1}^{n}a_{i}a_{j}P_{ij}^{*}}{A_{L}^{2}}$$

where *n* is the total number of green patches, P_ij_* is the maximum product probability of all possible paths between patches *i* and *j* (including direct dispersal between the two patches), *a_i_* and *a_j_* are the areas of the habitat at patches *i* and *j*, respectively, and *A_L_* is the total landscape area. The *PC* values are bounded (ranging from 0 to 1) and are defined as the probability of coincidence in a manner similar to the degree of coherence (Jaege, 2000).

(2) $$dPC_{k}=\frac{PC-PC_{remove,\;k}}{PC}\times 100\%$$

where *PC_remove,k_* is the overall possible connectivity of the remaining patch after removal of the ‘*k*’ green patch. *dPC_k_* measures the importance of the patches in maintaining the landscape connectivity through changes in the *PC* [46].

#### 2.2.2. The Thermal Variables for Describing Cooling Effect

To discuss the thermal condition of the three study areas, the thermal description variables were used as the air temperature (Ta), The difference of the Ta (ΔT) and the variation coefficient (CV) of Ta to reflect the cooling effect characteristics of the different green corridor patterns. Ta of green corridors were obtained by the ENVI-met 4.4.5 simulation results. The ΔT values and the CV of Ta were calculated as below.

(1) The difference of the Ta (ΔT)

The ΔT values was calculated by the average temperature inside the green corridors of each typical pattern minus the average temperature of the entire region based on the simulation results of the three study areas. The ΔT algorithm was used to analyse the cooling changes of the three typical green corridor patterns during the different time periods of a day.

(2) The variation coefficient (CV)

The coefficient of variation (CV) data of temperature in the internal region of green corridors was studied, which was used to reflect the dispersion of Ta values in each pattern of green corridors. Through the analysis of CV value changes of three green corridor patterns, it can be seen that the fluctuation variation effect of air temperature with the various spatial types of green corridors at the holistic block level. The formula for calculating coefficient of variation is as follows [47]:

(3) $$\mathrm{CV}=\frac{SD}{MN}$$

where CV is the coefficient of variation; *SD* is the standard deviation; *MN* is the average of each index.

### 2.3. ENVI-Met Dynamic Simulation

The spatial distribution data of the three blocks were extracted from the ArcGIS software using the high-definition aerial images of each study area. In the process of spatial simulation modelling, spatial integration and simplification of the relations of the internal elements were adopted for a holistic control of the design condition method from the urban design. The purpose of the simplified design was to reduce the disturbance of too many surrounding building details and internal spatial configuration impact on the microclimate environment of the green space, which makes the research highlight more the typical spatial structure and morphology of the green corridor pattern influencing the single cooling effect.

This study was simulated via ENVI-met 4.4.5, which was accessed on 24 June 2019. The weather data in this study were obtained from the weather station of Shanghai Hongqiao Airport on the Weather Underground website [48]. The initial input weather parameters are listed in Table 2, where the T and humidity values are the averages for the entire day of 24 June 2019. The initial values of Ta and RH were input as the hourly background corresponding values of the weather station of Shanghai Hongqiao Airport on 24 June 2019. The average value calculated using a simple forcing function was adopted. The mean temperature was obtained as 24.51 °C. The highest temperature at 14:00 reached 27.78 °C. The initial wind direction was southeast, which has the highest frequency during summer in Shanghai and the wind speed was 5.53 m/s at 10 m altitude.

In the modelling setting process, the influence of the 3D spatial composition of vegetation on the microclimate was set as the control variable. The criterion was that the vegetation was configured as a unified arbour and grass and the allocation ratio of arbour to grass was 1:30 plants/m^2^. Based on this basic criterion, relevant configuration parameters were determined (Table 3). Each grid was distributed using a two-layer structure of plant covers with trees and grasses composed of a 10 m high and 6 m wide tree canopy and 0.5 m of high grass.

### 2.4. BRT Model and Marginal Effect

The BRT model was developed by Elith et al. in 2008 [49]. Compared with the conventional regression model, the BRT model has certain advantages. It has strong adaptability to datasets and can deal with both continuous and categorical data. In addition, it is not sensitive to multiple linearity, does not need to consider the multiple collinearity problem faced by multiple linear regression analysis and can reflect the interaction of variables. Furthermore, the BRT model is a self-learning method based on a classified regression tree, which uses a boosting technology to fit multiple decision trees to determine the optimal model. It can compensate for the weak prediction ability of a single-tree model to the greatest extent [50]. In terms of the output results, the relative influence (or contribution) of each variable is scaled such that the sum adds up to 100. The larger the number, the greater the correlation with the dependent variable and the greater the impact contribution to the dependent variable [51]. The BRT model can also simulate the ME of independent variables and reflect their contributions to the dependent variables at different threshold intervals.

The BRT model was used to analyse ME changes of the multidimensional spatial factors of green corridors to the cooling effect in this study. Marginal effects are popular in some disciplines (e.g., economics), which refer to the new output or income by continuously increasing the input of a certain factor when other inputs are fixed [52]. In fact, marginal effect is a good illustration that output and input are not proportional; as long as the input exceeds a certain margin, output will disappear. The statistic calculated from the results of the existing fitting model, which represents the influence of the change of the independent variable on the change of the dependent variable. In this study, the marginal effect changes between each index and Ta can provide the impact threshold and correlation characteristics of each spatial morphological factor on air temperature by BRT model analysis.

The model was executed using the dismo package of the R language. The dependent variable was the Ta of the green corridors simulated by ENVI-met. The independent variables were the spatial indices of the green corridors. The model parameters were set as follows: the complexity of the decision tree was 5, the learning rate was 0.01, the segmentation ratio was 0.5 and the data type has a Gaussian distribution. The model extracted 50% of the data for analysis and 50% for training. Cross-validation was carried out 10 times to estimate the number of optimal trees. The contribution rate of each index of the BRT operation can determine the importance of the dependent variables to the difference in the Ta distribution. The ME changes of each spatial index were used to analyse the cooling impact threshold and correlation characteristics of each influencing factor.

### 2.5. The Study Framework

**Figure 4:**
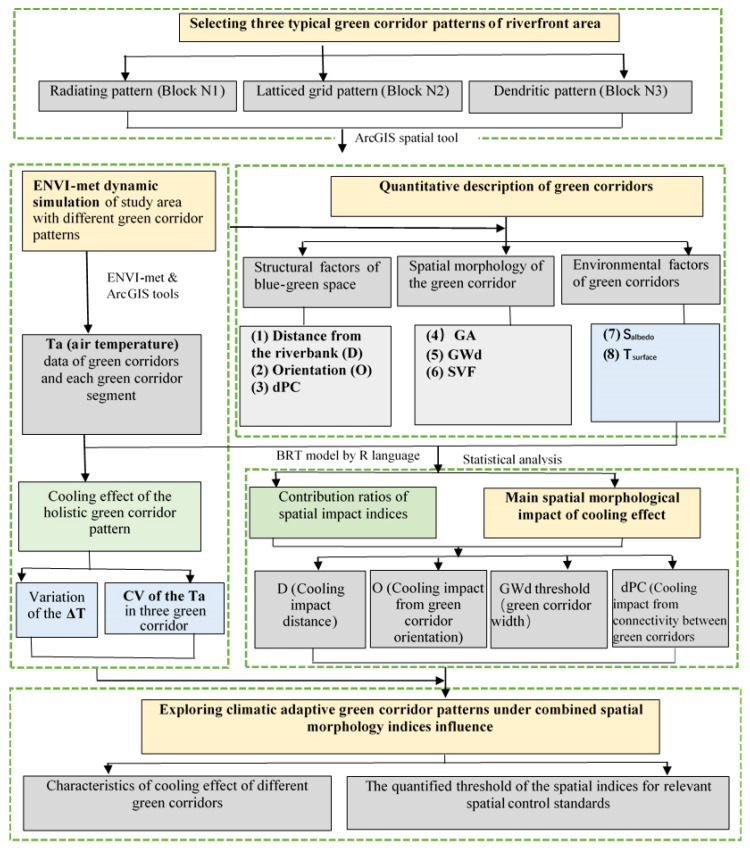
Flow chart of the overall study.

The framework of this study is shown in Figure 4. The study area contained three cases with different types of green space network patterns in riverfront urban blocks. The typical process for cooling effect studies involves four major steps: (1) Three-dimensional spatial model construction using the ENVI-met software and a microclimatic simulation. The spatial patterns were chosen based on satellite data combined with regulatory urban design data to identify the distribution of green corridors. The ENVI-met simulation results provided Ta values and the model physical spatial variables (Sky view factor (SVF), Tsurface and Albedo). (2) Quantitative description of green corridors. The climate-related spatial description variables were selected and calculated from three three aspects, namely, the structural factors of blue-green space, spatial morphology and environmental factors of green corridors. The network structure level to the individual spatial morphology factors (attribution data, spatial analysis, proximity analysis) by ArcGIS Guidos Toolbox, as well as to calculate the dPC index by Conefer 2.6 (by Santiago Saura and Josep Torné, at the Polytechnic University of Madrid and the University of Lleida, Spain). (3) BRT modelling analysis and statistical analysis of the results. Correlation determinations between the cooling effect and different types of spatial description variables were processed to obtain the contribution ratio of each factors’ impacts on the cooling effect. In addition, the detailed main individual spatial index affecting the Ta distribution under compound of multi-morphology factors. (4) Exploring climatic adaptive green corridor patterns under combined spatial morphology indices influence. For enhancing the cooling intensity of the blue-green corridor, the final step was clarified the differences in the cooling characteristics of different green corridors and explained the quantified threshold value of the spatial indices for relevant spatial control standards.

## 3. Result

### 3.1. Cooling Effect of the Holistic Green Corridor Pattern

**Figure 5:**
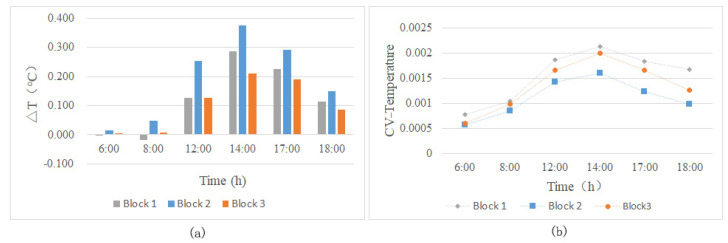
(**a**) Variation of the ΔT values and (**b**) CV of the Ta values for the three green space network patterns during typical diurnal hours.

The air temperature difference (ΔT) of the three studied green corridor patterns in the three blocks are significantly different (Figure 5a). Based on the ΔT changes of the three green corridor systems from 06:00 to 18:00 in the diurnal period, it can be observed that the cooling values gradually increase from 06:00 to 14:00. At 14:00, the ΔT values reach the maximum and then decrease gradually. Block N2 has the best cooling effect in the diurnal time, while the effect of block N1 is slightly better than that of block N3.

The variation coefficient (CV) was used to reflect the dispersion of Ta values in the green corridors in each study area (Figure 5b). Block N1 has green corridors in multiple directions, which are connected with the blue-green corridor in the south and can lead to a cold source airflow from the river surface. The connected green corridors are relatively wider and show large fluctuations in the CV of Ta. The green corridor with a large width exhibits the largest change in internal temperature at each time point. The cooling effect of block N2 is relatively stable. This green corridor pattern has the characteristics of good connectivity and the fluctuation of its internal Ta is the smallest. Block N3 comprises mostly the north–south corridors with a concentrated distribution and poor connectivity with the few east–west corridors. However, it has a relatively greater width (just larger than block N1) and its internal Ta fluctuation is better than that of block N2 and slightly lower than that of block N1.

### 3.2. Contribution Ratios of Spatial Impact Indices to the UCI of Green Corridors

Owing to the differences in the spatial characteristics of each block and the layout of the green corridor patterns, the contribution ratios of the spatial factors to the cooling effect are different. Through the BRT analysis between the established related indices of the riverfront green corridor system and the Ta value distribution in the green corridors, the values of the importance of the spatial indices of green spaces in different waterfront blocks were obtained (Table 4). (1) In block N1, D (42.77%), T_surface_ (33.75%) and GWd (8.1%) are the most important impact indices. (2) In block N2, D (28.23%), GWd (19.85%), T_surface_ (14.49%) and S_albedo_ (10.15%) are the most important impact indices and GA (9.27%) and dPC (8.62%) also have a relatively important impact on the cooling effect of green corridors. (3) In block N3, D (28.46%), T_surface_ (21.58%), dPC (14.29%) and GWd (13.11%) are the most important impact indices.

From the perspective of the impact factor classification, it can be observed that the interactions of the structural factors of blue-green space, spatial morphology and spatial environmental factors of green corridors produce the cooling effect of each green corridor pattern in the riverfront district.

(1) According to the relative importance of the structural factors of blue-green space, the D index shows a particularly important contribution to Ta in all three blocks. The dPC index of green corridors is more important in block N3. As all green corridors in block N1 have high connectivity and small numerical differences, the importance of the relative changes as a green space structural index is not shown. The orientation of the green corridor is a categorical variable, including only four values and it plays an important role to a certain extent.
(2) According to the relative importance of the spatial morphology index, GWd and GA are the factors describing the space scale of the green corridors, which account for a high proportion of contribution ratios among all factors. Because the linear morphology of green corridors is the main component of the green space pattern, it can be seen that the effect of GWd is more important than that of GA.
(3) According to the relative importance of environmental indicators, the Ta inside the green space is still greatly affected by the difference in the T_surface_ distribution. However, under the same design criteria, there are still differences in the distribution proportions of soft and hard surfaces in the internal spaces of the green corridors. The morphological changes in the surrounding building environment also affect the T_surface_ in the edge area of green corridors. These spatial environmental factors are closely related to the Ta distribution.

The simulation model of the relevant surrounding building environment and vegetation layout was pre-controlled to reduce the impact of too many uncontrollable environmental spaces on Ta and weaken the cooling impact caused by the differences in the morphological and structural factors of green corridors. Therefore, the data change ranges of SVF and S_albedo_ in this study are relatively small, indicating a small impact on the Ta values of the green corridors. This study also did not conduct a correlation analysis on the effect of these two indices.

### 3.3. Morphological Impact of Cooling Effect in Green Corridors

#### 3.3.1. Cooling Impact Distance of Blue-Green Corridor

The UCI of the blue-green space has a certain synergistic effect on the distance. From the changes in the ME between the D and Ta of each green corridor at different locations, it is necessary to determine the numerical significance of the following important inflection points of the ME:

(1) Within a certain distance, the ME of the three blocks shows a decreasing regular pattern.
(2) The ME reaches a zero value and the total utility reaches its highest value. This distance is considered as the largest cooling utility position for the blue-green synergistic effect.
(3) The ME decreases to a negative value, which indicates that this distance is still affected by the cooling effect of the belt blue-green space. However, its cooling effect no longer decreases with an increase in distance but changes in the opposite correlation. Based on this change, combined with the scatter diagram (Figure 5) of the green space temperature distribution at different locations in each green space corridor, we found that there exists a superposition effect of the cooling effect. Although the ME attenuation of the belt blue-green space exists within a certain range, the green corridor system creates a greater cooling effect in a special distance range. The superposition of the two types of UCI makes the Ta of green corridors in this special location lower than that in the green space near the riverbank.
(4) When the river cooling effect is basically zero and only the cooling island created by the green space system itself exists, a large inflection point in the marginal curve is observed. The position of the lowest attenuation value of the curve is no longer affected by the distance from the riverbank. This distance can be regarded as the maximum impact distance of the blue-green synergy function, namely the synergy cooling threshold of the blue-green space.

**Figure 6:**
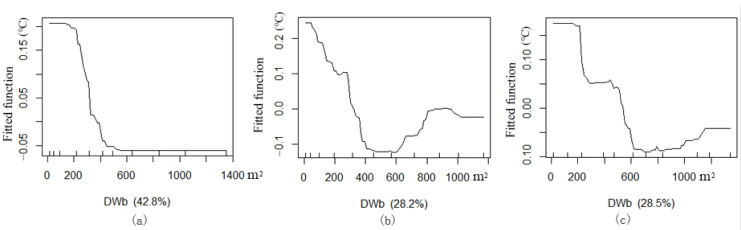
Marginal effect (ME) curve via boosted regression tree (BRT) between the distance from the water body (D) and T values of green corridors: (**a**) block N1, (**b**) block N2 and (**c**) block N3.

The cooling effect of the green corridor space in the three blocks shows slightly different impact ranges (Figure 6). (1) In block N1, the maximum total effect of the blue-green synergy cooling effect should be at the zero value of the marginal curve, approximately 380 m away from the riverbank. The maximum impact distance, i.e., the cooling threshold, is approximately 600 m (Figure 6a). (2) In block N2, the maximum value of the blue-green synergy cooling effect, namely the zero value of the marginal curve, is approximately 350 m away from the riverbank. The cooling threshold is approximately 600 m and the cooling intensity is relatively large (Figure 6b). (3) In block N3, the maximum value of the blue-green synergy cooling effect is approximately 560 m away from the riverbank and the cooling threshold is approximately 750 m (Figure 6c). Obviously, the green corridors with a north–south orientation in block N3 are conducive to the conveyance of cold air into the block and to the formation of cold air corridors.

**Figure 7:**
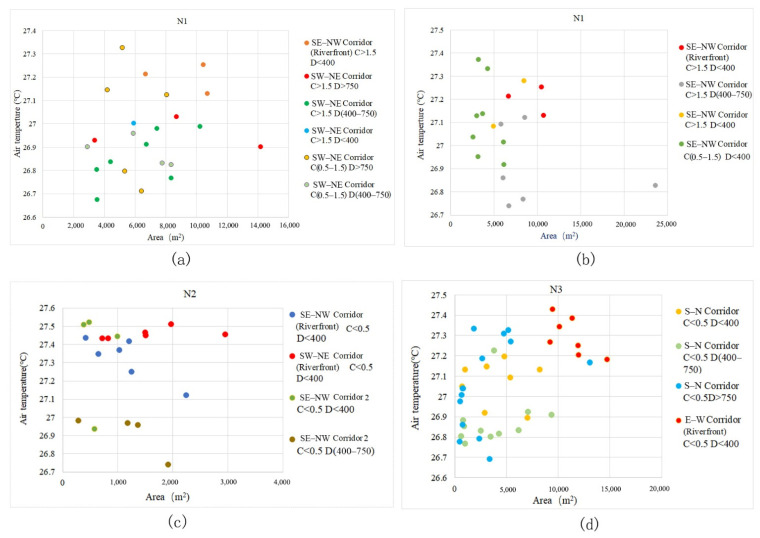
Scatter diagrams of green corridor segments with different D value grades and their corresponding Ta distributions. (**a**) In block N1, the distribution of Ta corresponding to green corridor D with the same WS–EN orientation indicates that when the dPC values of green corridors have the same grade, the values are more than 0.5 and the most corresponding T values in the low-temperature location belong to D values between 400 m and 750 m. (**b**) In block N1, with the same ES–WN orientation, the green corridor segments with a range of D from 400 m to 750 m also have the lowest Ta values. (**c**) In block N2, with the same ES–WN orientation, the D factor significantly affects the Ta distribution. When D is within 400 m, the cooling is attenuated with an increase in distance. When the D values of the green corridor segments are within 400–750 m grade with the same dPC and orientation, the internal Ta values in the green corridor segments are also the lowest. (**d**) In the green corridors of block N3 with an S–N orientation, the relationship between D and Ta still indicates the characteristic that the internal Ta value is low when D is within the 400–750 m grade.

Under the same level of other spatial impact factors (connectivity and orientation), we selected such green corridor segments with D changes and analysed the general characteristics of the cooling effect. As D increases, the Ta values of green corridors with different area scales change, as displayed in the scatter diagram (Figure 7).

General characteristic 1: The Ta values of green corridors that are not located in the area affected by the blue-green space are mostly higher than those of the green corridors located in the blue-green synergy impact area. The same grade of C > 1.5 is adopted, namely, the green corridors with good connectivity. The green corridors with D > 750 m grade almost attained the highest Ta values. Those with D < 400 m and D located in the range of 400 m to 750 m have a cooling island effect better than that of 750 m.

General characteristic 2: The lowest Ta values of the green corridors in the riverfront district occur between 400 m and 750 m. For the same C grade, the changes in the D grade of green corridors with two different orientations (Figure 7a–d) all show that in the building area near the riverbank, the Ta values of the belt green spaces are higher than those in green corridors with D values between 400 and 750 m and similar connectivity and orientation. In other words, when D is in the middle grade, the cooling island effect in the riverside area is the highest. Based on the ME curves of the three blocks (Figure 6), the maximum total effect of blue-green synergy cooling appears at approximately 400 m, which also shows that the transmission of cold air from the southern water body and the exchange of cold air within the green space are relatively complex. Under the superposition and synergy of the waterbody cooling island and the cooling island of the green space, the green corridor with a certain distance from the river achieves the lowest Ta.

#### 3.3.2. Cooling Impact from Green Corridor Orientation

In the R regression relationship between the spatial multi-variable factors and Ta distribution, the orientation of the green corridors has a certain effect. In the process of quantifying the orientation variables, the category values were determined to be of four types, resulting in a small proportion of importance. Our research aims to determine how the orientation of green corridors can adapt to climate change and produce a better cooling effect to the internal space.

**Figure 8:**
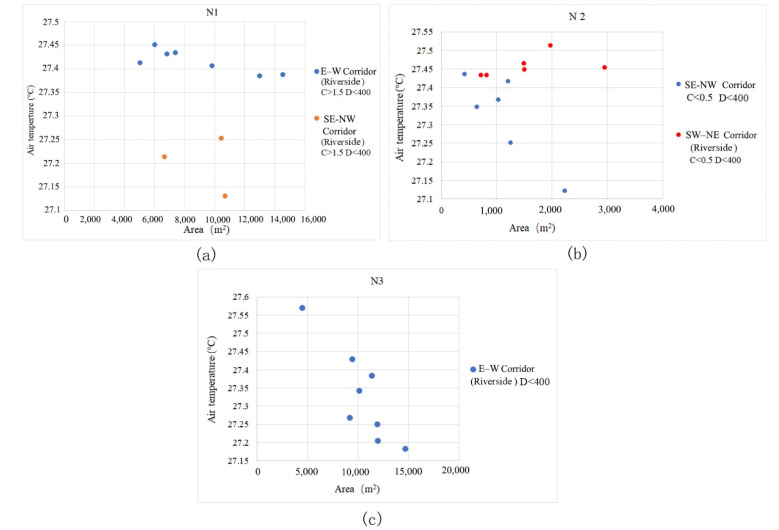
Scatter diagrams of Ta distribution with different composition segments corresponding to the orientation factors in the belt green space along the river channel. (**a**) Ta distribution of the riverside green corridor with different orientations in block N1: The Ta in the segments of the riverside green corridor with the SE–NW orientation is significantly lower than that in the E–W segments. (**b**) Ta distribution of the riverside green corridor with different orientations in block N2: The Ta in the segments of the riverside green corridor with the SE–NW orientation is considerably lower than that in the NE–SW segments. (**c**) Ta distribution of the riverside green corridor in block N3: The green corridor have an E–W orientation. Compared with the first two blocks with orientation changes in the river channel, a regular pattern of interaction between Ta and GA is obvious. The larger the GA of green corridor segments, the lower the Ta value in the internal green space.

The riverside green corridor and green corridors in the built environment have different cooling effects. A riverside green corridor combined with a river corridor produces a source of cold air, so its cooling intensity obviously affects the green corridor system in the downwind direction. Green corridors in the built environment are paths for conveying and exchanging the air temperature to achieve the cooling effect. We separated the two types and analysed the correlation between the corridor orientation and Ta to show the different impact characteristics in the green corridors. Combined with the spatial scale (GA values), connectivity degree (dPC) and the D condition of each green corridor, the influences of the orientation factors were compared and analysed (Figure 8).

The river channels of the three blocks are all located in the south and the river width is approximately 50 m. Owing to the difference in natural river channel orientation, the internal segments of the riverfront green corridors have a Ta difference that is greatly affected by the corridor orientation (Figure 8). In general, the Ta values in the interior space of the riverfront green corridor parallel to the prevailing wind direction are lower than those in other orientations. In block N3, the GA of the riverside green corridor is large and its orientation is relatively straight. The Ta of the E–W riverfront green corridor shows a higher GA index correlation. The larger the GA, the lower the Ta values in the corridor. In blocks N1 and N2, owing to the effect of the partial segment change in river orientation, the correlation between GA and Ta is complex. The Ta with two different orientations of the riverfront green corridors in block N2 is completely different and the SE–NW orientation has a larger cooling effect.

**Figure 9:**
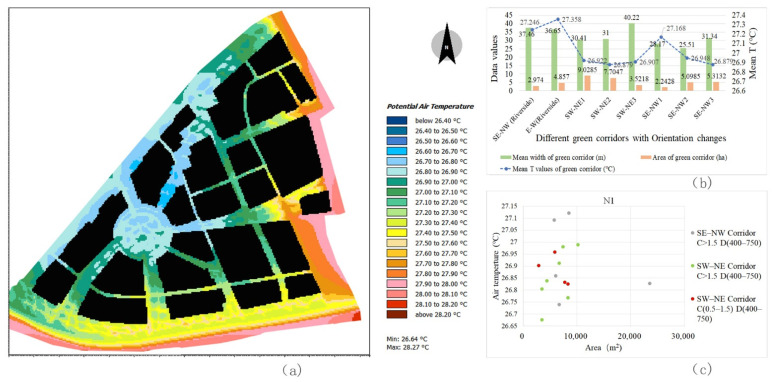
Ta distribution status of green corridors with different orientations at 14:00 and 1.5 m height in block N1 and comparison of the Ta difference of each green corridor segment. (**a**) Spatial distribution of Ta using ENVI-met simulation. (**b**) Comparison of mean temperatures of eight green corridors inside the block. The green corridor number is shown in Figure 2a. It can be seen that the green corridors with an SE–NW orientation have a lower Ta distribution. (**c**) Comparison of the Ta scatter distributions of two groups of green corridors with different orientations, the same grade of D factor and similar connection degree. It can be noticed that most Ta values of the green corridors with an SE–NW orientation are lower than those in the SW–NE oriented corridors. There are two outliers in the north location (these two green corridor segments are very narrow, independent of the radiating green space pattern and are independently located on the eastern edge of the block).

**Figure 10:**
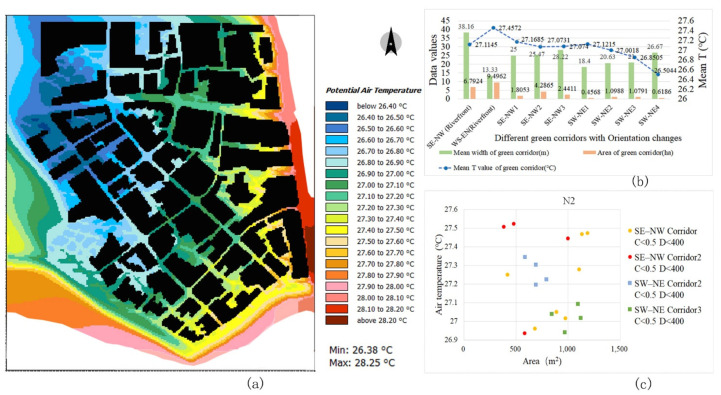
Ta distribution status of green corridors with different orientations at 14:00 and 1.5 m height in block N2 and comparison of the Ta difference of each green corridor segment. (**a**) Spatial distribution of Ta using ENVI-met simulation. (**b**) Comparison of mean temperatures of nine green corridors inside the block. The green corridor number is shown in Figure 2b. It can be seen that the blue-green corridors downwind of the SE–NW orientation have a significant impact on the Ta of the connected green corridors with an SW–NE orientation in the riverfront district. This blue-green spatial orientation factor affected the holistic cooling characteristics of the region such that the Ta values of the green corridors with an SW–NE orientation are mostly lower than the internal temperatures of the green corridors with an SE–NW orientation. (**c**) Comparison of Ta scatter distribution of two groups of green corridors with different orientations, the same grade of D factor and similar connection degree. It can be observed that most of the internal Ta of green corridors with an SW–NE orientation (marked by square points) are located below the internal Ta of green corridors with an SE–NW orientation (marked by circular points), which are relatively low.

**Figure 11:**
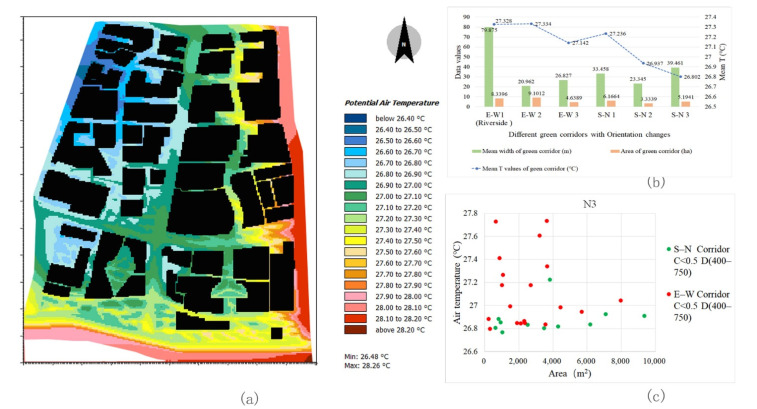
Ta distribution status of green corridors with different orientations at 14:00 and 1.5 m height in block N3 and comparison of the Ta difference of each green corridor segment. (**a**) Spatial distribution of Ta using ENVI-met simulation. (**b**) Comparison of mean temperature of six green corridors inside the block. The green corridor number is shown in Figure 2c. It can be observed that the Ta values of the S–N oriented green corridors with a small angle and with the summer monsoon wind direction are mostly lower than the internal Ta values of the E–W oriented green corridors. (**c**) Comparison of Ta scatter distributions of two groups of green corridors with different orientations, the same grade of D factor and similar connection degree. It can be seen that most of the internal Ta of green corridors with an S–N orientation (marked by green points) are located below the internal Ta of green corridors with an E–W orientation (marked by red points).

By comparing the cooling intensity of the riverfront green corridors in the three blocks, it can also be observed that the orientation factor has a significant impact on the Ta values. The cooling effect of block N2 is the highest (Figure 9b). The average width of its riverfront green corridor is 29.8 m, but the mean temperature inside it is the lowest, that is, 27.15 °C (Figure 10b). The average width of the riverfront green corridor of block N1 is approximately 37 m and the internal mean temperature of the green corridor is 27.36 °C (Figure 9b). The average width of the riverfront green corridor in block N3 is the largest (approximately 80 m) and the internal average temperature is 27.33 °C (Figure 11b). Although the green space width of block N3 is large, the synergy cooling effect of the blue-green corridors is obviously not the best. This shows that the SE–NW orientation corridors provide a low Ta value.

Cooling differences of green corridors with different orientations in the riverfront district.

Based on the orientation types of the green corridors identified in the previous process, the spatial cooling status of green corridors with different orientations in the riverfront district were analysed in detail. The cooling characteristics of each orientation affecting the Ta distribution can be summarised as follows:

(1) In block N1, SE–NW1 is a special location in the green corridor system, as it is a relatively independent green corridor on the outer edge, which is not related to the radiating structure of the main system. The internal Ta values of the other two SE–NW oriented green corridors have little differences compared to those of the three SW–NE oriented green corridors and the mean Ta of the SE–NW3 green corridor is the lowest (Figure 9a,b). However, by combining the GA and width factors, the scales of the two SE–NW oriented green corridors become smaller than those of the SW–NE corridors. The SE–NW3 green corridor has the smallest GA and its Ta value is the lowest among all green corridors with different orientations (Figure 9a,b). Combined with the scatter diagram in Figure 9c, compared to some green corridor segments with similar GA values, the Ta values in the SE–NW3 green corridors are lower than those in the SW–NE corridors.
(2) In block N2, an interesting phenomenon is that the orientation impact of the green corridors is completely opposite to that in block N1. The Ta values of the SW–NE oriented green corridors are significantly lower than those of the SE–NW corridors (Figure 10b). In the scatter diagram, the other spatial influencing factors at the same grade and the impact of different orientation factors are also consistent with this observation. In the partial green corridor segments with similar GA (Figure 10c), the cooling effect of green corridors with an SW–NE orientation is better than that with an SE–NW orientation. By combining with the Ta distribution map of the dynamic simulation (Figure 10a), we found that in block N2, the SE–NW river corridor and its riverside green corridor have a significant impact on the cooling of the three green corridors with an SW–NE orientation, showing the influence of cooling attenuation far away from the riverbank. This can reasonably explain the inconsistent finding of the orientation factor impact in block N2.
(3) In block N3, the Ta values of the green corridors with an S–N orientation are lower than those with the E–W orientation (Figure 11a–c). The green corridor width of the S–N orientation is the largest among the three blocks and the GA of each independent segment is large as well. However, there is a little difference in the mean Ta between blocks N3 and N1 (Figure 11a,b). The result of block N1 indicates that the SE–NW orientation has an obvious advantage relative to the cooling effect in green corridors with different orientations. However, combined with the cooling distance of the three blocks, the distance achieving the maximum cooling effect in the green corridor with an S–N orientation is 650 m, which is greater than that with an SE–NW orientation of approximately 400 m. Similarly, the maximum cooling threshold distance is 750 m, which is also greater than the distance threshold of 600 m corresponding to the green corridor with an SE–NW orientation.

In the analysis of the characteristics of the green corridors in the riverfront district, it can be noticed that the belt blue-green space is a cold source with a synergistic function of the cooling island during the high-temperature diurnal period in summer. The green corridor of the surrounding built environment helps to convey cold air through transmission and air exchange over a longer distance, resulting in a large-area cooling effect. In general, the cooling effect of the green corridor with an SE–NW orientation is the highest and the advantage of the green corridor with an S–N orientation is that the cooling effect distance is longer.

#### 3.3.3. GWd Threshold

**Figure 12:**
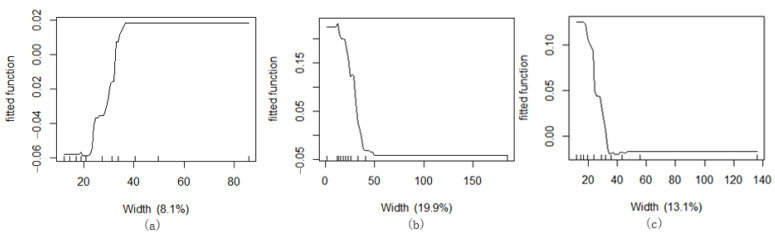
ME curve of BRT between the GWd factor and Ta of green corridors: (**a**) block N1, (**b**) block N2 and (**c**) block N3.

Based on the ME between the width of the green corridors and Ta values, the corresponding T values of the green corridor width in the three blocks show different cooling effect characteristics (Figure 12). The maximum GWd value of the cooling ME is at the first inflection point of the curve, where the curve shows a gentle or downward tendency. The maximum total cooling effect is the zero value of the marginal curve.

In block N1, the GWd distribution is uniform and relatively larger and the relationship between the GWd and Ta distribution shows mostly positive effects, but the effect growth range is very small. In other words, with an increase in GWd, the ME of cooling is increasing slowly. The ME becomes stable when the width increases to nearly 40 m. The total cooling effect of the green corridors reaches maximum at approximately 33.8 m width (zero value of ME).

In blocks N2 and N3, the GWd data of green corridors are diverse and the two ME curves between Ta and GWd present a relatively consistent regular pattern. For block N2, the GWd is approximately 20 m when the cooling effect of ME appeared inflection point, namely, after this inflection point, the change effect of the dependent variable Ta caused by each increase of one unit of the independent variable GWd begins to decrease and the GWD value at this position is the optimum choice for cooling effect; when the GWd is greater than 20 m, the cooling effect continues to increase with increasing GWd. However, the increasing width of the green corridor continues to provide more cooling effect. When the GWd of the green corridor reaches 30 m (zero value of ME), the total cooling effect is the largest. When the GWd of the green corridor in block N3 is approximately 25 m, the cooling effect is the optimum state and the total cooling effect of the green corridor is the largest at approximately 35 m.

**Figure 13:**
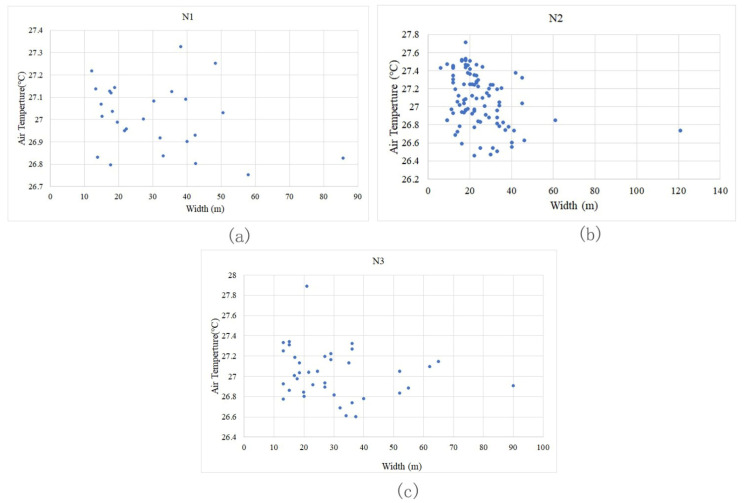
Scatter diagram of the GWd factor and its corresponding Ta distribution: (**a**) block N1, (**b**) block N2 and (**c**) block N3. Although the Ta values of different green corridor segments in the three blocks are affected by other spatial factors, such as dPC, the GWd and Ta of green corridors are correlated, i.e., if the width of green corridors is larger, the Ta is lower.

From the GWd data and Ta value distribution of the green corridor segments at different locations in the scatter diagram of the three blocks (Figure 13), it can be seen that the relationship between the GWd and Ta in the three blocks is negatively correlated. The Ta changes of the green corridors in block N1 affected by the change in GWd are gentle. The range of Ta values caused by the increase in GWd is relatively smaller. Compared with other blocks, the GWd in block N2 changes significantly such that the change range of Ta is relatively large and the negative correlation between them is obvious. In block N3, the Ta change range is between those of blocks N1 and N2 with the width variation range increased.

#### 3.3.4. Cooling Impact of the dPC Factor

**Figure 14:**
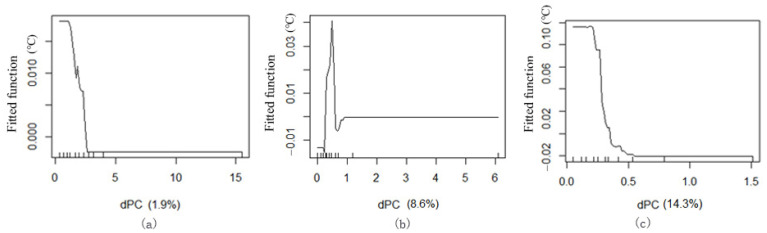
ME curve of BRT between the dPC factor and Ta of green corridors: (**a**) block N1, (**b**) block N2 and (**c**) block N3.

The ME curve between the green space network connectivity (dPC) and Ta values indicates that the dPC index considerably affects the spatial temperature distribution of green corridors in the three blocks. The results show that most of the data intervals between them have a negative correlation, that is, the greater the dPC, the lower the Ta (Figure 14).

Most of the green corridors in block N1 have high dPC values. From the comparison of the cooling effect range, the influence of dPC on the Ta in the green corridor segments is the smallest among the three blocks. When the dPC is 1.5, the ME on Ta reaches its maximum. When the dPC is greater than 1.5, it has a particularly significant negative correlation with an increase in dPC and the dPC value range of block N2 is large. The arc of the ME curve is very large, indicating that the Ta changes significantly with an increase in dPC. When the dPC reaches 0.5, the ME of cooling reaches the maximum. The green corridors in block N3 are mainly N–S oriented corridors. The dPC values of most green corridors are very low and the majority are within 0.5. The data tendency is very similar to that of block N1. When the dPC is lower than 0.5, it also has a significant negative correlation with Ta.

Therefore, two important interval values of connectivity, i.e., 0.5 and 1.5, were identified. These values show the important variation characteristics of Ta, which becomes the interpretable reason for grading of the dPC factor.

**Figure 15:**
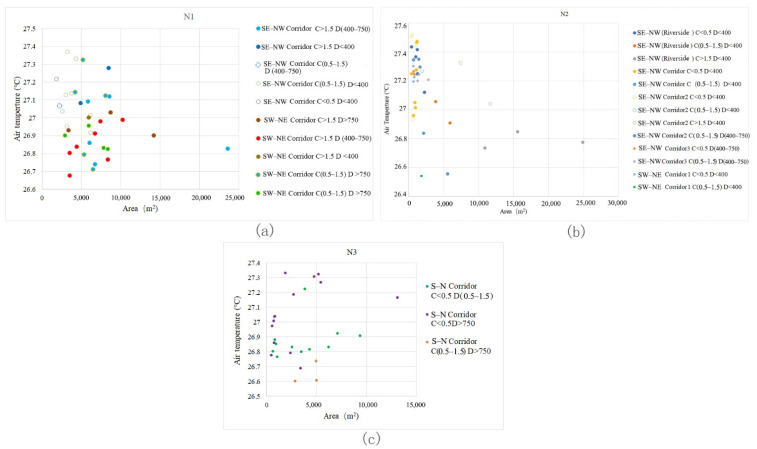
Scatter diagram of spatial connectivity and corresponding temperature distribution of green corridors. (**a**) In block 1, most of the T values corresponding to the green corridors with C > 1.5 are below the connectivity grade where C is 0.5–1.5 and C < 0.5, that is, the connectivity is large and the corresponding Ta value is low. (**b**) In block 2, the relationship between connectivity and Ta in the riverside green corridor segments is particularly significant. The higher the grade of C values, the lower the Ta values. In addition, compared with the group data of two grades of green corridors with C > 1.5 and C of 0.5–1.5, most of the Ta values corresponding to C > 1.5 are in the middle and lower part of the chart and most of the Ta values corresponding to C < 0.5 are in the upper part of the chart. (**c**) In block 3, the Ta values of green corridors with C < 0.5 are mostly in the upper part of the chart and the C values of the green corridors are in the range of 0.5–1.5, which are located in the low Ta values.

From the scatter diagram of the connectivity grade change and Ta value distribution of green corridor segments at different locations in the three blocks, it can be seen that with the increase in connectivity, the internal Ta of green corridors decreases (Figure 15). In block N1, the dPC values are generally large, the Ta distribution in the green corridors is relatively uniform and the overall change range is small. In block N2, the dPC of green corridors is relatively better and the overall internal Ta shows an obvious downward tendency with an increase in dPC. In block N3, the N–S oriented green corridors are the main components. They have a connectivity value range of less than 1.5, which is smaller than that in block N2. In this block, there are only two grades of connectivity data, i.e., dPC grades of less than 0.5 and 0.5–1.5. The green corridors with high connectivity are characterised by low temperatures.

## 4. Discussion

### 4.1. Characteristics of Cooling Effect of Different Green Corridors

Green corridors with different morphological characteristics have different functions in achieving the cooling effect of the riverfront area. The key to maximising the cooling effect of green corridors is to optimise the advantages of different spatial structures and morphologies of green corridors. The essence of the cooling effect of the spatial structure pattern is to promote cold air exchange and air circulation in the green corridors. The type of green corridor morphology conducive to alleviating the impact of high temperatures in the riverfront area in summer needs to be identified. Through the enhancement in the cooling intensity of the blue-green corridor and suitable transmission of the cold source air by green corridors in the built environment, we can strengthen cold air exchange and air circulation in the green corridor system, which is a favourable development model for green corridors in the riverfront area. This result can be explained by the following aspects, which can also clarify the differences in the cooling characteristics of different green corridors.

(1) Layout of leeward green corridors for direct cooling effect

In the analysis of the cooling effect of green corridors with different orientations for the three blocks, the leeward green corridors have the characteristics of low temperature. In blocks N1 and N3, the cooling effect of the green corridors with an SE–NW orientation is better than that of the SW–NE orientation. The S–N orientation of green corridors is better than the E–W orientation and the cooling distance of the S–N green corridors in block N3 is up to 750 m. Along the leeward direction, when the corridors are wide enough, the air circulation and exchange of cold air more directly influence the cooling effect under the action of wind.

(2) Enhancing the cold source effect of the blue-green corridor for effective cooling

In block N2, the river passing through the south has an ES–WN orientation segment. The riverside green corridor with this orientation shows a spatial distribution change in distance attenuation for the cooling of the three green corridors with the SW–NE orientation, resulting in the Ta of the green corridors with an SW–NE orientation being lower than that with an SE–NW orientation. In other words, although the SW–NE orientation is not leeward, the leeward blue-green corridor with an ES–WN orientation provides low cold source air. The cold air diffuses, flows and exchanges through the SW–NE orientation corridors and the mean Ta of green corridors with this orientation is lower than that of the green corridors with a downwind SE–NW orientation. This further proves that the air exchange and circulation of cold air in the green corridors are more important. Therefore, the blue-green corridor serves as a cold source. Under the pressure difference between the cold source air and the hot air of the built-up area, a rational layout of the air circulation and exchange channel is an important measure for enhancing the cooling effect of the entire area.

(3) Dual superposition cooling effect of green corridors in the built-up area

The D of each green corridor segment in the three blocks has a significant impact on the Ta distribution and its importance proportion is the highest among the spatial factors related to Ta. The proportion for block N1 reaches 42.77% and it reaches more than 28% for the other two blocks. However, the D factor does not produce the minimum Ta distribution in the riverfront green corridors. The Ta of the riverside green corridor with the E–W orientation is higher than that of the leeward green corridors connecting the blue-green corridor. The cooling of the water body should be spread horizontally in the wilderness area, so that the open space itself at a certain distance from the riverbank will have a regular pattern of distance attenuation. According to this characteristic, the riverside is a low-temperature distribution area close to the river. However, in the riverfront building space, the Ta of the extended green corridors becomes low. This shows that the green corridor system in the riverfront built-up space conveys the cold source air and at the same time, its own cooling effect produces a superposition effect at a certain distance from the river channel. The green corridors connecting the riverside blue-green corridor are an effective structural pattern for cooling.

(4) Green corridor perpendicular to the leeward direction for improving cooling by increasing connectivity

The grid pattern of green corridors is the optimal combination pattern and its cooling effect is greater than that of the other two patterns of independent green corridors. The grid green corridor network pattern of block N2 (when the connectivity of the spatial structure is above 0.5) shows that connectivity plays a significant role in cooling. The WS–EN orientation of the blue-green corridor or the E–W orientation of connected green corridors also assists in improving the connectivity of various corridors with the ES–WN and S–N orientations, resulting in the overall best cooling effect of the blue-green corridors in the block.

### 4.2. Implications for Climatic Adaptive Design in the Riverfront Area

A holistic and suitable layout of blue-green spaces and surrounding areas can provide a better thermal environmental quality with waterbodies and green composite riverfront areas. As an important channel of open space and bioclimate, green corridors play an important role in the circulation of external and internal airflows and affect the thermal environment of the entire area near the river. In the process of constructing the green space network formed by the green corridors in the waterfront area, this study adopted the ME curve to present the maximum threshold of the spatial indices. These threshold values can provide a basic reference for relevant control standards of waterfront planning and design.

In this study, it was found that the wide green corridors in the area close to the south waterbody can effectively serve as a cold source for the waterbody and riverbank green corridor. In blocks N1 and N2, the average width of the south riverside green corridor is approximately 30–40 m, the width of the local green corridor is approximately 50–55 m and the influence distance of the blue-green synergistic cooling island effect is approximately 600 m. In block N3, under the influence of the riverside green corridor of approximately 100 m width, the cooling influence distance is 750 m.

The relationship with the prevailing wind direction should be considered when setting the orientation of green corridors in the riverfront district. The green corridor orientation should be consistent with the prevailing wind direction of the summer wind, or perpendicular to the river channel direction as far as possible, which is conducive to the internal air circulation of the green corridors in summer. A suitable distribution of green corridor orientation is conducive to introducing cold air over the river and expanding the influence range of the cold source through air passage. In this study, we found that the green spaces in the ES–WN and S–N directions can transmit the cold source of water to a longer distance and achieve a better cooling effect.

However, different green corridors have different cooling effects. The E–W riverside green corridor achieves the effect of cold airflow and diffusion by improving the connectivity index of the green corridors. The appropriate setting of SW–NE and E–W oriented green corridors enhances the connectivity of the SE–NW and S–N green corridors in the block. These green corridors have different cooling and cold air transmission modes but jointly promote a comprehensive and coordinated cooling effect of the blue-green space.

When the width of the green corridor in the riverfront built-up area is 20–25 m, the cooling ME is the highest under the condition of limited land use. When the width is 30–35 m, the total cooling effect is the optimum. In planning and designing the layout of interconnected green corridors in the riverfront area as a cold air transmission channel, an economic width scale of 20–25 m can be considered. When conditions permit, 30–35 m of green corridors can also be arranged to achieve the best overall cooling effect.

### 4.3. Differences from Previous Studies

The cooling effects of collaborative blue-green corridor patterns originate from a very complex and comprehensive ecological process. Most of the existing studies have discussed the microclimatic effects of UGSs on a macroscale. Moreover, previous studies mainly used a separate perspective to study the green space and water body and only few studies have considered their synergistic microclimatic effects [53]. The correlation between the blue-green spatial morphology and microclimatic effect is not necessarily linear. It is difficult to capture the complex and nonlinear relationships of variables using parameter regression methods. The machine learning algorithm does not pre-set the relationship model in correlation research, which provides an idea for the optimisation of correlation research. This research method can not only identify the relative importance of each influencing factor but also simulate the ME of independent variables to comprehensively consider the interaction and importance of each variable.

Combined with the urban riverfront district, through the analysis of the blue-green spatial multi-morphology factors that specifically affect the temperature distribution, this study demonstrated that it can meet the constraint standard for climatic adaptative planning and layout of blue-green corridor systems and provide guidance on specific spatial factors for local practical construction.

### 4.4. Limitations of This Study

Because the riverfront green corridor model has many limitations in the selection conditions of the field case area, the selection of the case area only aimed to maintain the similarity of the site as far as possible, such as the site area, building development intensity, green space ratio and water body located in the south. In the research process, the urban design method was adopted, the plant allocation proportion was consistent and each green space layout had strict layout criteria. These pre-set conditions ensured less interference from the spatial environment and the 3D morphological characteristics of vegetation to focus on the impact of changes in spatial layout planning conditions and related indices. However, owing to the complexity and diversity of the internal composition of the actual site, the cooling effect of the green corridor network of the three patterns was affected by many influencing factors and the number of samples was slightly small. Under possible conditions in the future, we can increase the case scenario of the simulation and further study the role of other influencing factors to provide more reference strategies for the construction of a riverfront green space network in the future.

In addition, the study only investigated the cooling effect during a typical summer high-temperature period of the blue-green corridors under the influence of many spatial variables. The impact of vegetation composition, position and morphology of the water body are also important spatial factors in the riverfront area. Thus, the cooling characteristics of other more extensive green networks with different structures and morphologies in the riverfront area need to be further explored.

## 5. Conclusions

The microclimate of the riverfront area of three typical green corridor patterns in Shanghai was simulated using ENVI-met and its influence on the cooling effect was analysed from three aspects: structural factors of blue-green space, spatial morphology and environmental factors of green corridors. The ME and threshold analyses of the BRT model were conducted for various spatial factors and Ta distribution data. It was found that the four spatial factors, namely the distance from the riverbank, corridor orientation, width and connectivity degree of green corridors in the riverfront area can be combined to form a suitable structural pattern that enhances the effect of UCI.

In the spatial description index of green corridors affecting the riverfront case area, the relationship between the distance from the riverbank and the spatial temperature distribution of the green corridors shows a gradient change regular pattern. By selecting 14:00, in which the green corridor system has the largest cooling effect during the diurnal period in summer, the impact distance of the blue-green cooling island effect is transmitted to 600–750 m. The green corridors corresponding to the prevailing wind direction of the summer monsoon are more conducive to the long-distance transmission of the cold source southward of the urban district.

As a cold source transmission channel, the riverfront green corridors play an important role in the cooling island effect. The green corridors with a direction parallel to or roughly the same as the prevailing wind direction (S–N) have good cooling effects. Their cooling effects are greater than those of the E–W orientation. The cold air interactions between the green corridors with the SW–NE and SE–NW orientations produce the optimal cooling pattern of the green corridor network.

The impact of the width factor of the green corridor on the air temperature shows a regular pattern, i.e., the larger the corridor width, the lower the air temperature. When the width of the green corridors is 20–25 m, the ME of cooling is the largest. At 30–35 m, the overall ME of cooling is the highest and tends to be stable.

The dPC of the green corridors is negatively correlated with the temperature distribution. In the range of 0.5–1.5, it can significantly improve the cooling island effect of the green corridor. When the dPC value reaches 1.5, its ME reaches the maximum.

There are still many specific spatial factors affecting the distribution characteristics of the thermal environment that need to be discussed in detail. In general, the BRT model provides a comprehensive comparison of the influences of all factors on the cooling effect. This research method is worth introducing and applying to more quantitative research at the mesoscale or microscale and the combination of 3D dynamic simulation and GIS analysis can be used to thoroughly compare and analyse the study process.

## Figures and Tables

**Table 1:** Spatial index to describe the structure and morphology of green corridors.

Impact Variables	Selected Index	Definition and Description
Structural factors of blue-green space	Distance from the riverbank (D)	The distance between the geometric centre of each green corridor segment and the riverbank, which is used to represent the influence of the blue space on the cooling effect of the green space.
	Orientation (O)	The orientation of the green corridor is used to reflect the consistency between the linear green corridor and dominant wind direction.
	Decreased probability connectivity (dPC)	The connectivity index characterising the contribution of each green space in the connectivity degree to the overall green space network.
Spatial morphology of the green corridor	GA	The surface area that the green space occupies.
	GWd	The green corridor width is the cross-sectional width.
	SVF	The ratio of the sky hemisphere visible from the ground (not obstructed by buildings, terrain, or trees).
Environmental factors of green corridors	S_albedo_	This index is the ratio of the surface reflection flux to the incident solar radiation flux on the surface of the green space.
	T_surface_	The ground surface temperature of the covered area of green space.

**Table 2:** Initial input values of weather parameters for the simulation model.

Input Parameters	Ta (°C)	Wind Orientation	Wind Speed (m/s)	RH (%)	Roughness
Value	24.51	135°	5.53	66.46	0.01

**Table 3:** Main modelling parameter and configuration file settings used for ENVI-met simulations.

	Input Category and Parameter	Value(s) Used
Modelling parameter	Size of grid cell (m) (dx, dy, dz)	6, 6, gradient height; the lowest grid dz is 3 m.
	Number of nesting grids	5
Configuration file setting	Simulation date	Date: 24 June 2019; Starting time: 06:00 Simulated time: 24 h
	Meteorological conditions (basic data)	Temperature range: 21–28 °C Wind speed (10 m): 5.53 ms^−1^ Wind direction: 135° (from the west)
		3D plant: privet with 10 m high and 5 m wide tree canopy
		Simple plant: Grass 50 cm aver, dense
		Roughness length: 0.01
	Material for nesting grids	Soil A: loamy soil; Soil B: loamy soil
	Default wall material	Moderate insulation
	Default roof material	Moderate insulation

**Table 4:** Relative importance of the green corridor indices for each simulation model.

Spatial Indices for Riverfront District	Relative Importance of Predictor Variables (%)		
	Block N1	Block N2	Block N3
D	42.77	28.23	28.46
dPC	1.93	8.62	14.29
O	4.53	4.17	2.36
GWd	8.1	19.85	13.11
GA	2.42	9.27	6.90
SVF	4.66	5.22	8.80
S_albedo_	1.83	10.15	4.50
T_surface_	33.75	14.49	21.58

## Data Availability

We choose to exclude this statement.

## Abbreviations

SCE	Synergistic cooling effect
UHI	Urban heat island
UCI	Urban cooling island
UBI	Urban green space cool island
Ta	Air temperature (°C)
ΔT	The difference between T and the mean T of the entire study area
CV	Coefficient of variation
BRT	Boosted regression trees
ME	Marginal effect
Block N1/N2/N3	Block no. 1/no. 2/no.
E-W	East-west orientation
S-N	South-north orientation
SW-NE	Southwest -northeast orientation
SE-NW	Southeast-northwest orientation
C	Connectivity
PC	Probability of connectivity
dPC	The decrease probability connectivity
D	Distance from river bank
O	Orientation
GA	Green space area
GWd	Green corridor width
SVF	Sky view factor
S_Albedo_	Surface albedo
T_surface_	Surface temperature
